# Diagnosis of Myotonic Dystrophy Based on a History of Grip Myotonia in a 21-Year-Old Woman Undiagnosed for Five Years: A Case Report

**DOI:** 10.7759/cureus.85558

**Published:** 2025-06-08

**Authors:** Hiroki Maita, Tadashi Kobayashi, Takashi Akimoto, Hiroshi Osawa, Hiroyuki Hanada

**Affiliations:** 1 Development of Community Healthcare, Hirosaki University Graduate School of Medicine, Hirosaki, JPN; 2 General Medicine, Hirosaki University School of Medicine and Hospital, Hirosaki, JPN; 3 Emergency, Disaster and General Medicine, Hirosaki University Graduate School of Medicine, Hirosaki, JPN

**Keywords:** creatine kinase, delayed finger opening, history-taking, hyperckemia, myotonic dystrophy, physical examination

## Abstract

Myotonic dystrophy (DM) is an inherited neuromuscular disorder characterized by myotonia and progressive muscle weakness. A 21-year-old female patient presented with a one-month history of fever, myalgia, and a threefold increase in creatine kinase (CK) levels following a diagnosis of coronavirus disease 2019. The patient had undergone hemithyroidectomy at 14 years of age and was being followed up with regular blood testing, which revealed that she had slightly elevated CK levels (232-265 U/L) but consistently normal thyroid hormone levels. At the initial visit, detailed history recording revealed that since attending high school, the patient had occasionally experienced difficulty in opening her gripped hand. Physical examination confirmed grip myotonia, and apart from the elevated CK levels (333-488 U/L; CK isozyme MM 98%), blood tests revealed no obvious abnormalities. Genetic analysis revealed CTG trinucleotide repeat expansion, confirming a diagnosis of DM type 1. DM should accordingly be considered as a differential diagnosis in cases of an unexplained elevation in CK levels, particularly in younger patients. Detailed history recording and physical examination of myotonia should be performed if DM is suspected.

## Introduction

Myotonic dystrophy (DM) is the most prevalent form of muscular dystrophy, affecting one in 3,000-8,000 individuals globally [1]. It is an autosomal-dominant inherited disorder characterized by skeletal muscle weakness, myotonia, abnormal cardiac conduction, and cataracts [2]. The severity of symptoms ranges from severe and congenital to almost asymptomatic, and it is often unrecognized and undiagnosed, particularly in mild cases [2]. According to a Japanese study, the average duration between initially becoming aware of symptoms and visiting a medical institution is three years, with this duration being >5 years in 23.5% of patients [3]. Symptoms at the time of the first visit are mainly reduced grip strength, stiffness of the fingers, lower limb disability, and complications during pregnancy [3]. Dyspnea has also been reported as a rare presentation [4]. Despite the detection sensitivity of myotonia being approximately 90%, physicians should proactively identify symptoms in patients with mild or unrecognized myotonia [5]. Timely identification of the disease can lead to prompt interventions, thereby improving patient quality of life [4].

Although elevated levels of creatine kinase (CK) detected in blood tests are typically the initial finding during consultation [2], this may be an incidental finding in patients with no or few muscle-related signs or symptoms, and accordingly, managing these patients can be clinically challenging [6]. Moreover, a number of non-neuromuscular diseases, including thyroid disease, electrolyte abnormalities, and drug-related conditions, are similarly associated with an elevation in CK levels [6]. Patients with DM may generally present with a slight-to-moderate elevation in CK levels, and in asymptomatic patients with hyperCKemia, achieving an accurate diagnosis of DM may take a long time [7].

Herein, we describe the case of a patient with a history of grip myotonia referred for a thorough examination of unexplained elevated CK levels, leading to a diagnosis of DM.

This article was previously presented in part as a poster presentation at the 15th Annual Conference of the Japan Primary Care Association on June 9, 2024.

## Case presentation

A 21-year-old woman with a history of thyroid tumor surgery presented to our hospital with elevated CK levels, fever, and myalgia. She had episodes of slow running; however, there was no apparent disease in her childhood, and no family history of genetic or neurological disorders. At 14 years of age, the patient had undergone hemithyroidectomy for early-stage papillary thyroid cancer and was subsequently followed up with regular blood tests, including thyroid function tests, and the results of this consistently remained within the normal range. The CK levels during the follow-up period were found to be slightly elevated, ranging from 232 to 265 U/L. She worked as a nursing assistant at a hospital and led an unrestricted daily life. At two months prior to the visit, she had experienced a slight fever and back pain, and, accordingly, visited a local orthopedic clinic. However, the underlying cause was not determined, and her back pain subsequently resolved spontaneously. After one month, she acquired a coronavirus disease 2019 infection and subsequently developed prolonged fever, transient neck muscle pain, and elevated CK levels (333-488 U/L; CK isozyme MM 98%). At the initial visit, her back and neck pain had already improved, and no abnormal vital signs (axillary temperature, 36.6°C; blood pressure, 104/67 mmHg; pulse rate, 66 beats/min) were recorded. Other than episodes of myalgia, the patient had no cardiopulmonary, ocular, or articular symptoms. Furthermore, she had no relatives presenting with similar symptoms and had no history of regular medication use. Detailed history recording revealed that since attending high school, the patient had occasionally experienced difficulty in opening her gripped hand, and a physical examination confirmed grip myotonia. No other neurological abnormalities, such as muscle atrophy or weakness, were observed. The patient was examined for muscular dystrophy, polymyositis, viral myositis, drug-induced myositis, hypothyroidism, mitochondrial disease, periodic paralysis of the extremities, and paraneoplastic syndromes. However, apart from the elevated CK levels, blood tests revealed no obvious abnormalities (Table 1). Similarly, urinalysis, electrocardiogram, and positron emission tomography-computed tomography indicated no abnormalities. Genetic analysis confirmed the diagnosis of DM type 1 based on the finding of an elongation (≈400 times) of a CTG trinucleotide repeat in the DM protein kinase (DMPK) gene. Electromyography was not performed because the genetic test result was definitive for DM type 1. Approximately one year after diagnosis, the patient’s activities of daily living have been maintained with continued care by a DM specialist.

**Table 1:** Hematological and biochemical test results

Variable	Reference range	Upon presentation
White blood cell count (/μL)	3300−8600	6200
Red blood cell count (×10^4^/μL)	386−492	413
Hemoglobin (g/dL)	11.6−14.8	11.7
Mean corpuscular volume (fL)	83.6−98.2	89.1
Mean corpuscular hemoglobin (pg)	27.5−33.2	28.3
Platelets (/μL)	158,000−348,000	353,000
Erythrocyte sedimentation rate (1 h/2 h) (mm)	3−15 (1 h)	38/59
Albumin (g/dL)	4.1−5.1	4.6
Blood urea nitrogen (mg/dL)	8−20	7
Creatinine (mg/dL)	0.46−0.79	0.62
Sodium (mmol/L)	138−145	141
Potassium (mmol/L)	3.6−4.8	3.6
Chloride (mmol/L)	101−108	103
Calcium (mg/dL)	8.8−10.1	9.6
Aspartate aminotransferase (U/L)	13−30	33
Alanine aminotransferase (U/L)	7−23	25
Alkaline phosphatase (U/L)	38−113	73
Lactate dehydrogenase (U/L)	124−222	208
Total bilirubin (mg/dL)	0.4−1.5	0.4
Gamma-glutamyl transferase (U/L)	9−32	29
Creatine phosphokinase (U/L)	41−153	398
C-reactive protein (mg/dL)	0−0.14	0.18
Ferritin (ng/mL)	12−60	16.6
Thyroid-stimulating hormone (μIU/mL)	0.50−5.00	2.25
Free triiodothyronine (pg/mL)	2.30−4.00	2.65
Free thyroxine (ng/mL)	0.90−1.70	1.06
Anti-nuclear antibody	<40	40
Anti-aminoacyl-tRNA synthetase antibody	Negative	Negative

## Discussion

This case report highlights two key points, the first of which is that DM should be considered as a differential diagnosis in patients with an unexplained elevation of CK levels. The absence of symptoms or few symptoms in patients with elevated CK levels could be associated not only with primary neuromuscular diseases (muscular dystrophy, metabolic disorders, mitochondrial disorders, and inflammatory myopathies) but also with various non-neuromuscular diseases (e.g., endocrine disorders, electrolyte disorders, and muscle trauma) and medications [6]. Persistent asymptomatic hyperCKemia has been considered to be a mild or early stage of myopathy [8], indicating the importance of muscle and genetic testing for patients presenting with these features. The European Federation of Neurological Societies guidelines [9] recommend biopsy for asymptomatic patients with elevated CK levels who present with any of the following characteristics: (1) abnormal (myopathic) findings on electromyography, (2) CK levels ≥ 3 times the upper normal limit, (3) age < 25 years, or (4) exercise-induced pain or exercise intolerance. The patient described herein had undergone surgery for a thyroid tumor at the age of 14 years and was being followed up with regular blood testing. It is plausible that a diagnosis of DM in this young patient with an unexplained elevation of CK levels may have been achieved earlier if the threshold for detailed examination had been lowered using the aforementioned guidelines.

Second, when DM is suspected, taking into consideration a history of myotonia (a characteristic feature of DM) is important. However, patients may not recognize this as a disease-related symptom, and it can thus be readily overlooked unless suspected by health care providers [2]. In Japan, a screening method for DM comprising myotonia, muscle weakness of the hands and fingers, neck muscle weakness (difficulty in raising the head), trunk muscle weakness (difficulty in getting up), and a family history of the disease has been developed. It is anticipated that this will facilitate a timely detection of DM by non-neurologists. Among these items, myotonia (fingers, jaw, and eyelids) has been established to provide the best diagnostic accuracy, with a sensitivity of 87.4%-92.2% and specificity of 68.8%-78.8% [5]. Although our patient was aware of grip myotonia that had persisted since attending high school, the complaint was only confirmed via a detailed history recording. Accordingly, as an initial step in DM diagnosis, the identification of unrecognized history and physical findings is important.

As a limitation of this case report, it is not possible to specify the influence of coronavirus or influenza virus infection or vaccination on symptom onset and CK levels. The coexistence of other conditions that may cause myositis should be interpreted with caution.

## Conclusions

DM should be considered as a differential diagnosis in cases of an unexplained elevation in CK levels, particularly in younger patients. If DM is suspected, detailed history recording and physical examination of myotonia should be performed. Early diagnosis, along with appropriate intervention and support, may contribute to an improved quality of life for patients.
